# Protocol to study the immune profile of syngeneic mouse tumor models

**DOI:** 10.1016/j.xpro.2024.103139

**Published:** 2024-06-14

**Authors:** Sayuri Miyauchi, Kei-ichiro Arimoto, Mengdan Liu, Yue Zhang, Dong-Er Zhang

**Affiliations:** 1 Moores Cancer Center, University of California, San Diego, La Jolla, CA 92037, USA; 2 School of Biological Sciences, University of California, San Diego, La Jolla, CA 92037, USA; 3 Department of Pathology, University of California, San Diego, La Jolla, CA 92037, USA

**Keywords:** single cell, flow cytometry, cancer, immunology

## Abstract

Flow cytometry, single-cell RNA sequencing, and other analyses enable us to capture immune profiles of the tumor microenvironment. Here, we present a protocol to characterize the immune profile of tumor-bearing mice. We describe steps for establishing mouse models and preparing single-cell suspensions from tumor tissue and other immune-related organs, which can be further analyzed by flow cytometry and other omics assays. We then detail procedures for staining, flow cytometry analysis, and phenotyping of the immune cell populations.

For complete details on the use and execution of this protocol, please refer to Miyauchi et al.^1^

## Highlights

• Generalized preparation of single-cell suspensions from tumors, organs, and tissue
• Immunophenotyping of tumors and immune organs by flow cytometry analysis
• Investigate the effects of treatments or genes/proteins on the tumor microenvironment

## Abstract (editor highlights)

Publisher’s note: Undertaking any experimental protocol requires adherence to local institutional guidelines for laboratory safety and ethics.

## Graphical abstract

**Figure:**
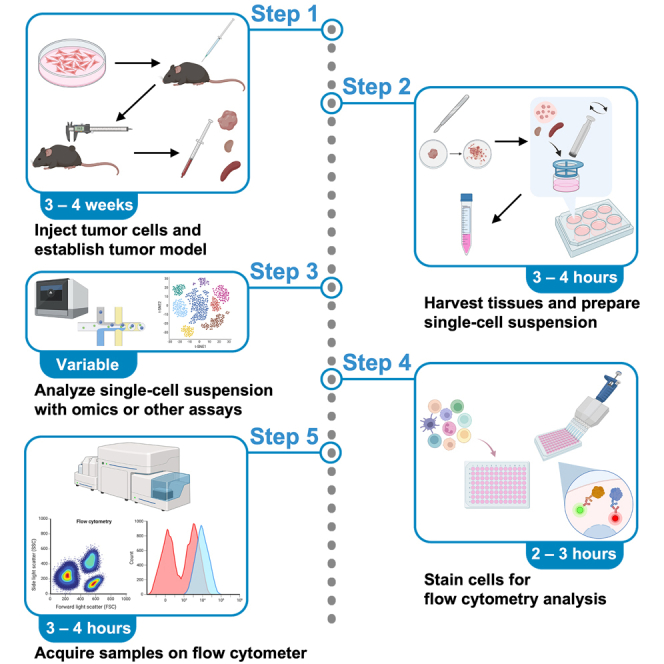

## Before you begin

This protocol describes processes in investigating anti-tumor immune response by analyzing immune cell profiles in syngeneic mouse tumor models. Here, we analyzed B16F10 melanoma, LLC lung carcinoma, and MC38 colon carcinoma in C57BL/6 mice. However, this protocol could be applied broadly to tumor types to obtain single-cell preparations by optimizing the tissue dissociation step. We have also used this protocol for E0771 mammary cancer in C57BL/6 mice and 4T1 mammary cancer in BALB/c mice. The staining panels for flow cytometry analysis can be modified depending on the immune populations of interest or the flow cytometer configuration.

### Institutional permissions

Institutional permission should be obtained for animal studies. All animal-related work in our studies was approved by the Institutional Animal Care and Use Committee of the University of California San Diego (S07271). All mice were housed and bred in a specific pathogen-free vivarium at Moores Cancer Center at the University of California San Diego accredited by the American Association for the Accreditation of Laboratory Animal Care.

#### Cell culture

**Timing: 5–7 days**

1. Revive cells from frozen vials and culture them in the appropriate culture medium.

***Note:*** The composition of the cell culture medium for B16F10, LLC, and MC38 can be found in the Materials and equipment setup section.

2. Passage the cells at least once before using them for injection to ensure cells are growing well.

***Note:*** Cells without at least one passage might not be fully revived and can cause slower *in vivo* growth. On the other hand, too many passages can cause changes in the characteristics of cell lines. Using cells at passages 2 or 3 is ideal.

3. Use cells at ∼80% confluency for injection.

***Note:*** Overconfluent cells might not be in a healthy condition and can lead to lower engraftment rates *in vivo* and/or slower *in vivo* growth.

#### Staining panel design for flow cytometry analysis

**Timing: variable**

The staining panels for flow cytometry in this protocol were designed for NovoCyte Advanteon Flow Cytometer (Agilent Technologies) equipped with 3 lasers (violet, blue, and red) and a total of 19 detectors. Staining panels need to be designed based on the configuration of your flow cytometer and your proteins/markers of interest.

***Note:*** Examples of staining panels for myeloid cells and lymphoid cells are shown in Table 1 and Table 2, respectively.

4. Know the configuration of your flow cytometer: number of lasers, number of detectors, and range of wavelengths.
5. Design your staining panel.
  a. Use brighter fluorochromes for rare/lowly expressed proteins and use dimmer fluorochromes for highly expressed proteins.
  b. If possible, use different lasers for markers of the same cell population. For example, Treg markers CD25 and Foxp3 are best detected using fluorochromes excited by different lasers. In the example lymphoid panel (Table 2), we used PE (excited by blue laser) for CD25 and APC (excited by red laser) for Foxp3.
  c. Test and optimize your staining panels.

**Table 1:** Antibody panel to identify myeloid cell populations

Antibody	Clone	Fluorochrome	Dilution	Product
(Viability dye)	-	Zombie Aqua	1:1000	BioLegend, Cat #: 423101

**Cell surface staining**				

CD45.2	104	Alexa Fluor 700	1:200	BioLegend, Cat #: 109822
CD11b	M1/70	PE-Cy5	1:500	BioLegend, Cat #: 101210
Gr-1	RB6-8C5	PE-Cy7	1:200	BioLegend, Cat #: 565033
F4/80	BM8	BV605	1:100	BioLegend, Cat #: 123133
Ly6C	HK1.4	APC	1:100	BioLegend, Cat #: 128016
Ly6G	1A8	FITC	1:100	BioLegend, Cat #: 127606
CD11c	N418	BV570	1:200	BioLegend, Cat #: 117331
I-A/I-E (MHC II)	M5/114.15.2	BV711	1:200	BioLegend, Cat #: 107643
CD3	17A2	BV650	1:100	BioLegend, Cat #: 100229
CD19	6D5	PE-Dazzle594	1:100	BioLegend, Cat #: 115554
CD80	16-10A1	BV421	1:100	BioLegend, Cat #: 104726

**Intracellular staining**				

CD206	C068C2	PE	1:100	BioLegend, Cat #: 141706

**Table 2:** Antibody panel to identify lymphoid cell populations

Antibody	Clone	Fluorochrome	Dilution	Product
(Viability dye)	-	Zombie Near-IR	1:1000	BioLegend, Cat #: 423106

**Cell surface staining**				

CD45.2	104	Alexa Fluor 700	1:200	BioLegend, Cat #: 109822
CD3	17A2	BV711	1:100	BioLegend, Cat #: 100241
CD4	RM4-5	BV570	1:500	BioLegend, Cat #: 100542
CD8a	53–6.7	PE-Cy5	1:200	BioLegend, Cat #: 100710
CD25	PC61	PE	1:100	BioLegend, Cat #: 102008
CD62L	MEL-14	PE-Cy7	1:100	BioLegend, Cat #: 104418
CD44	IM7	FITC	1:100	BioLegend, Cat #: 103006
NK1.1	PK136	BV605	1:100	BioLegend, Cat #: 108753
CD19	6D5	BV785	1:100	BioLegend, Cat #: 115543
B220	RA3-6B2	BV510	1:200	BioLegend, Cat #: 103248
CD11b	M1/70	PE-Dazzle594	1:500	BioLegend, Cat #: 101256
CD69	H1.2F3	BV650	1:100	BioLegend, Cat #: 104541
CD71	RI7217	BV421	1:100	BioLegend, Cat #: 113813

**Intracellular staining**				

Foxp3	FJK-16s	APC	1:100	Thermo Fisher Scientific (eBioscience), Cat #: 17–5773-82

## Key resources table

**Table:** 

REAGENT or RESOURCE	SOURCE	IDENTIFIER
**Antibodies**		

Rat monoclonal anti-mouse CD16/CD32 (Clone: 93) (1:50)	Thermo Fisher Scientific (eBioscience)	Cat #: 14-0161-82; RRID: AB_467133
Rat monoclonal anti-mouse CD3 (clone 17A2) Brilliant Violet 650 (1:100)	BioLegend	Cat #: 100229; RRID: AB_11204249
Rat monoclonal anti-mouse CD3 (clone 17A2) Brilliant Violet 711 (1:100)	BioLegend	Cat #: 100241; RRID: AB_2563945
Rat monoclonal anti-mouse CD4 (clone RM4-5) Brilliant Violet 570 (1:500)	BioLegend	Cat #: 100542 (also 100541); RRID: AB_2563051
Rat monoclonal anti-mouse CD8a (clone 53-6.7) PE-Cyanine5 (1:200)	BioLegend	Cat #: 100710 (also 100709); RRID: AB_312749
Rat monoclonal anti-mouse/human CD11b (clone M1/70) PE-Dazzle594 (1:500)	BioLegend	Cat #: 101256 (also 101255); RRID: AB_2563648
Rat monoclonal anti-mouse/human CD11b (clone M1/70) PE-Cyanine5 (1:500)	BioLegend	Cat #: 101210 (also 101209); RRID: AB_312793
Armenian hamster monoclonal anti-mouse CD11c (clone N418) Brilliant Violet 570 (1:200)	BioLegend	Cat #: 117331; RRID: AB_10900261
Rat monoclonal anti-mouse CD19 (clone 6D5) Brilliant Violet 785 (1:100)	BioLegend	Cat #: 115543; RRID: AB_11218994
Rat monoclonal anti-mouse CD19 (clone 6D5) PE-Dazzle594 (1:100)	BioLegend	Cat #: 115554 (also 115553); RRID: AB_2564001
Rat monoclonal anti-mouse CD25 (clone PC61) PE (1:100)	BioLegend	Cat #: 102008 (also 102007); RRID: AB_312857
Rat monoclonal anti-mouse/human CD44 (clone IM7) FITC (1:100)	BioLegend	Cat #: 103006 (also 103005, 103021, 103022); RRID: AB_312957
Mouse monoclonal anti-mouse CD45.2 (clone 104) Alexa Fluor 700 (1:200)	BioLegend	Cat #: 109822 (also 109821); RRID: AB_493731
Rat monoclonal anti-mouse/human CD45R/B220 (clone RA3-6B2) Brilliant Violet 510 (1:200)	BioLegend	Cat #: 103248 (also 103247); RRID: AB_2650679
Rat monoclonal anti-mouse CD62L (clone MEL-14) PE-Cyanine7 (1:100)	BioLegend	Cat #: 104418 (also 104417); RRID: AB_313103
Armenian hamster monoclonal anti-mouse CD69 (clone H1.2F3) Brilliant Violet 650 (1:100)	BioLegend	Cat #: 104541; RRID: AB_2616934
Rat monoclonal anti-mouse CD71 (clone RI7217) Brilliant Violet 421 (1:100)	BioLegend	Cat #: 113813; RRID: AB_10899739
Armenian hamster monoclonal anti-mouse CD80 (clone 16-10A1) Brilliant Violet 421 (1:100)	BioLegend	Cat #: 104726 (also 104725); RRID: AB_2561445
Rat monoclonal anti-mouse CD206 (MMR) (clone C068C2) PE (1:100)	BioLegend	Cat #: 141706 (also 141705); RRID: AB_10895754
Rat monoclonal anti-mouse F4/80 (clone BM8) Brilliant Violet 605 (1:100)	BioLegend	Cat #: 123133; RRID: AB_2562305
Rat monoclonal anti-mouse Foxp3 (clone FJK-16s) APC (1:100)	Thermo Fisher Scientific (eBioscience)	Cat #: 17-5773-82; RRID: AB_469457
Rat monoclonal anti-mouse Gr-1 (Ly6G/Ly6C) (clone RB6-8C5) PE-Cyanine7 (1:200)	BD Biosciences	Cat #: 565033; RRID: AB_2739049
Rat monoclonal anti-mouse I-A/I-E (clone M5/114.15.2) Brilliant Violet 711 (1:200)	BioLegend	Cat #: 107643; RRID: AB_2565976
Rat monoclonal anti-mouse Ly6C (clone HK1.4) APC (1:100)	BioLegend	Cat #: 128016 (also 128015); RRID: AB_1732076
Rat monoclonal anti-mouse Ly6G (clone 1A8) FITC (1:100)	BioLegend	Cat #: 127606 (also 127605); RRID: AB_1236494
Mouse monoclonal anti-mouse NK-1.1 (clone PK136) Brilliant Violet 605 (1:100)	BioLegend	Cat #: 108753 (also 108739, 108740); RRID: AB_2686977

**Chemicals, peptides, and recombinant proteins**		

RPMI 1640 medium, no glutamine	Thermo Fisher Scientific (Gibco)	Cat #: 21870076
Fetal bovine serum, USDA approved, heat inactivated	Omega Scientific	Cat #: FB-02
Penicillin-Streptomycin (10,000 U/mL)	Thermo Fisher Scientific (Gibco)	Cat #: 15140122
L-glutamine (200 mM)	Thermo Fisher Scientific (Gibco)	Cat #: 25030081
0.25% Trypsin 2.21 mM EDTA, 1X	Corning	Cat #: 25-053-CI
Trypan blue solution, 0.4%	Thermo Fisher Scientific (Gibco)	Cat #: 15250061
DPBS, no calcium, no magnesium	Thermo Fisher Scientific (Gibco)	Cat #: 14190144
HBSS (10X), no calcium, no magnesium, no phenol red	Thermo Fisher Scientific (Gibco)	Cat #: 14185052
HBSS, no calcium, no magnesium, no phenol red	Thermo Fisher Scientific (Gibco)	Cat #: 14175095
HBSS, no calcium, no magnesium	Thermo Fisher Scientific (Gibco)	Cat #: 14170112
ACK lysing buffer	Thermo Fisher Scientific (Gibco)	Cat #: A1049201
Bovine serum albumin, fraction V, cold-ethanol precipitated	Fisher Scientific	Cat #: BP1605
Ethylenediaminetetraacetic acid (0.5 M solution/pH 8.0)	Fisher Scientific	Cat #: BP2482
Sodium azide, crystalline, laboratory	Fisher Scientific	Cat #: S227I
Collagenase D from *Clostridium histolyticum*	Roche	Cat #: 11088882001
Percoll	Cytiva	Cat #: 17089101
Zombie Aqua Fixable Viability Kit	BioLegend	Cat #: 423101
Zombie NIR Fixable Viability Kit	BioLegend	Cat #: 423106
Brilliant stain buffer	BD Biosciences	Cat #: 566349
eBioscience Foxp3/transcription factor staining buffer set	Thermo Fisher Scientific (Invitrogen)	Cat #: 00-5523-00
UltraComp eBeads Plus compensation beads	Thermo Fisher Scientific (Invitrogen)	Cat #: 01-3333-42
ArC Amine Reactive Compensation Bead Kit	Thermo Fisher Scientific (Invitrogen)	Cat #: A10346
Corning Matrigel matrix	Corning	Cat #: 354234

**Experimental models: Cell lines**		

Mouse: B16F10	Dr. Michiko Fukuda (Sanford Burnham Prebys Institute)	RRID: CVCL_0159
Mouse: Lewis lung carcinoma (LLC)	National Cancer Institute	RRID: CVCL_4358
Mouse: MC38	Dr. Andrew Sharabi (University of California, San Diego)	RRID: CVCL_B288

**Experimental models: Organisms/strains**		

Mouse: C57BL/6J (wild type, 6–9 weeks, male and female)	The Jackson Laboratory	Strain #: 000664; RRID: IMSR_JAX:000664

**Software and algorithms**		

NovoExpress	Agilent Technologies	Link
FlowJo 10	BD Biosciences (FlowJo, LLC)	https://www.flowjo.com
GraphPad Prism 8	GraphPad	https://www.graphpad.com

**Other**		

BD Luer Slip Tip syringe with attached needle 25 G × 5/8 in., sterile, single use, 1 mL	Becton Dickinson	Cat #: 309626
Microvette 100 EDTA K3E, EDTA-coated blood collection tube	Sarstedt	Cat #: 20.1278.100
BD Luer-Lok Syringe sterile, single use, 5 mL	Becton Dickinson	Cat #: 309646
Fisherbrand Sterile cell strainers (70 μm)	Fisher Scientific	Cat #: 2-363-548
Fisherbrand Disposable soda-lime glass Pasteur pipets	Fisher Scientific	Cat #: 13-678-6B
Countess II FL automated cell counter	Thermo Fisher Scientific (Invitrogen)	N/A
BRANDplates microtitration plate, 96-well, pureGrade	BRAND GmbH + Co. KG	Cat #: 781601
NovoCyte Advanteon Flow cytometer	Agilent Technologies	N/A

## Materials and equipment

**Table:** Cell culture medium (for B16F10, LLC, and MC38)

Reagent	Final concentration	Amount
RPMI 1640	N/A	440 mL
Fetal Bovine Serum (heat-inactivated)	10%	50 mL
Penicillin-Streptomycin (10,000 U/mL)	100 U/mL	5 mL
L-Glutamine (200 mM)	2 mM	5 mL
**Total**	N/A	**500 mL**

[Store at 4°C for up to 3 months.]

**Table:** 100x Collagenase D solution

Reagent	Final concentration	Amount
Collagenase D	100 mg/mL	100 mg
PBS	N/A	1 mL
**Total**	N/A	**1 mL**

[Store at −20°C for up to 1 month.]

**Table:** 1x Collagenase D solution

Reagent	Final concentration	Amount
100x Collagenase D solution	1 mg/mL	0.5 mL
RPMI	N/A	49.5 mL
**Total**	N/A	**50 mL**

[Prepare on the day of the experiment and use immediately.]

**Table:** Stock isotonic Percoll (SIP)

Reagent	Final concentration	Amount
Percoll	90%	7.2 mL
HBSS 10x, clear	10%	0.8 mL
**Total**	N/A	**8 mL**

[Prepare on the day of the experiment and keep it at 20°C–25°C until use.]

**Table:** 40% Percoll (SIP)

Reagent	Final concentration	Amount
Percoll (SIP)	40%	3.2 mL
HBSS 1x, clear	60%	4.8 mL
**Total**	N/A	**8 mL**

[Prepare on the day of the experiment and keep it at 20°C–25°C until use.]

**Table:** 80% Percoll (SIP)

Reagent	Final concentration	Amount
Percoll (SIP)	80%	4 mL
HBSS 1x, red	20%	1 mL
**Total**	N/A	**5 mL**

[Prepare on the day of the experiment and keep it at 20°C–25°C until use.]

**Table:** Flow cytometry buffer

Reagent	Final concentration	Amount
HBSS 1x, clear	N/A	495 mL
BSA	0.5%	2.5 g
EDTA 0.5 M	2.5 mM	2.5 mL
Sodium azide 10%	0.05%	2.5 mL
**Total**	N/A	**500 mL**

[Store at 4°C for up to 6 months.]

***Note:*** Sodium azide helps to prevent shedding and internalization of cell surface antigens, but it also inhibits metabolic activities. Therefore, do not add sodium azide if cells are to be collected for functional assays (i.e. if sorted live cells will be used for downstream analysis).

**Table:** Fixable Live/Dead solution

Reagent	Final concentration	Amount
Zombie Fixable Viability Kit (fluorochrome of your choice)	1:1000	1 μL
PBS	N/A	1 mL
**Total**	N/A	**1 mL**

[Prepare on the day of the experiment and use immediately.]

**Table:** Fc blocking solution

Reagent	Final concentration	Amount
Anti-mouse CD16/32 antibody	1:50	10 μL
Flow cytometry buffer	N/A	490 μL
**Total**	N/A	**500 μL**

[Prepare on the day of the experiment and use immediately.]

**Table:** 1x Fixation buffer

Reagent	Final concentration	Amount
Fixation/Permeabilization Concentrate (4x) (Thermo, Cat # 00–5123)	1x	1 mL
Fixation/Permeabilization Diluent (Thermo, Cat # 00–5223)	N/A	3 mL
**Total**	N/A	**4 mL**

[Prepare on the day of the experiment and keep it at 4°C.]

**Table:** 1x Permeabilization buffer

Reagent	Final concentration	Amount
Permeabilization Buffer (10x) (Thermo, Cat # 00–8333)	1x	1 mL
Distilled water	N/A	9 mL
**Total**	N/A	**10 mL**

[Prepare on the day of the experiment and keep it at 4°C.]

## Step-by-step method details

### Part 1: Injection of tumor cells into syngeneic mice

**Timing: 2 h**

This section details how to inject tumor cells into syngeneic mice subcutaneously at the flank.

***Note:*** Cells should be cultured in different sizes of dishes or flasks depending on the desired cell number for your experiment. Solution volume should be scaled up or down accordingly. Here, the protocol describes the procedure for a 150 mm dish.

1. Culture cancer cell line in appropriate culture medium. (See before you begin, cell culture).
2. Trypsinize cells.
  a. Remove culture medium, wash cells with sterile PBS, and then remove the PBS.
  b. Add 2–4 mL of trypsin-EDTA for a 150 mm dish and incubate the dishes at 37°C for 3–5 min or until all the cells are detached from the culture dish. Optimal trypsin incubation times will vary on a per-cell line basis.
    ***Note:*** Add 1–2 mL of trypsin-EDTA for a 100 mm dish.
    ***Note:*** Dishes may be gently tapped to promote cell detachment during trypsin incubation. Cell detachment can be confirmed by visual inspection or with a microscope if not distinguishable by eye.
  c. Add 5 mL of fetal bovine serum (FBS)-containing medium to stop the trypsin reaction, and collect cell suspension in a sterile centrifuge tube.
  d. Centrifuge the cells for 5 min at 400–600 × g at 20°C–25°C.
3. Wash cells with sterile PBS.
  a. Remove the supernatant and resuspend the cells in PBS. Add PBS up to the maximum volume of the centrifuge tube.
  b. Centrifuge the cells for 5 min at 400–600 × g at 20°C–25°C.
  c. Remove the supernatant and resuspend the cells in PBS. Recommended cell concentration is 1 × 10^5^–1 × 10^7^ cells/mL for accurate cell counting.

***Note:*** If the cell suspension contains clumps, filtering the cell suspension is recommended to ensure a single-cell suspension.

4. Adjust the concentration of the cell suspension.
  a. Determine cell number by using an automated cell counter or hematocytometer.
  b. Adjust cell suspension to the desired concentration based on the cell counts.
  c. Keep the cell suspension on ice until use.

***Note:*** Typically, 1–5 × 10^5^ cells in 100 μL per mouse are appropriate for a subcutaneous flank model.^2^^,^^3^^,^^4^^,^^5^ In this protocol, 1 × 10^5^ cells of B16F10 or 5 × 10^5^ cells of LLC or MC38 in 100 μL were subcutaneously injected into the flank.

***Note:*** Adjust the volume of cell suspension per mouse according to your tumor model. For example, 25–30 μL would be appropriate for the tongue orthotopic tumor model.

***Note:*** Subcutaneous tumor models are easier for tumor cell implantation and tumor monitoring compared to orthotopic tumor models. However, ideally, the cell lines should be injected into the organ of origin to mimic the native tumor microenvironment. For example, E0771 and 4T1 cells are better to be injected into the mammary fat pad.

***Optional:*** If the injected cell lines show unstable growth, the addition of Matrigel at 50% of the final volume would help with *in vivo* growth.

5. Inject cells into mice.
  a. Put a mouse into an isoflurane chamber to anesthetize it.
  b. Shave the fur at the injection site.
  c. Load cell suspension into a 1 mL syringe with a 25G needle.
  d. Sanitize injection site by spraying with 70% ethanol or an equivalent sanitizing agent that is approved in your institution’s animal protocol.
    **CRITICAL:** Ensure that the mouse is under sedation during the injection by using a nose cone to avoid it waking up in the middle of the injection.
    ***Note:*** An ophthalmic ointment can be used to protect their eyes during sedation although it is not necessary to use it for a brief sedation.
  e. Insert the needle subcutaneously and slowly inject 100 μL of cell suspension.

### Part 2: Establishment of tumor-bearing mouse models

**Timing: 3–4 weeks**

This section describes the process of tumor monitoring and measurement.

6. Start measuring the tumors when they become palpable.

***Note:*** Tumors may take 5–8 days to become palpable depending on the cell line and the number of cells injected.

7. Measure tumor size every 2–3 days with a caliper until sacrifice. The longest diameter is “Length” and the transverse diameter is “Width”.
8. Calculate tumor volume using this formula: Tumor volume (mm^3^) = (Length × Width^2^)/2.

***Note:*** Measure tumors as frequently as required for their size as specified in your institution’s policy.

***Note:*** Sacrifice timing depends on the tumor model (growth speed). Depending on the scientific question you would like to address, it may be preferable to have multiple time points to capture certain anti-tumor immune responses and tumor microenvironments.

### Part 3: Dissection of mice and harvesting tissue

**Timing: 15 min per mouse**

This section explains how to obtain different tissues including blood, tumor, tumor-draining lymph nodes, and spleen from a mouse.

9. Euthanize mice by CO_2_ inhalation followed by cervical dislocation in accordance with IACUC policy. (Please follow your institution’s IACUC guidelines on animal euthanasia.).
10. Place the mouse on its back and spray it with 70% ethanol.
11. Open the thorax and expose the heart. Collect blood via cardiac punctures with a 25G needle syringe.

***Note:*** This step needs to be done quickly as the blood coagulates rapidly.

***Note:*** It is recommended to collect 50–100 μL of blood for flow cytometric analysis. The required volume varies depending on the downstream assay.

12. Place the blood in EDTA- or heparin-coated tubes.

***Note:*** Detach the needle from the syringe before expelling blood into EDTA/heparin tubes to avoid hemolysis.

***Note:*** Ensure that the volume of blood does not exceed the maximum, manufacturer-recommended volume of the collection tube. Excess volume causes blood clotting. Split into multiple tubes as necessary. Invert tubes to fully mix blood with EDTA or heparin.

13. Carefully dissect the mouse and harvest organs of interest.

***Note:*** The anatomical location of the tumor-draining lymph node varies depending on your tumor model. Inguinal lymph nodes are the tumor-draining lymph nodes for flank tumor models.

14. Keep the harvested organs in flow cytometry buffer on ice while preparing to proceed to the next step.

***Note:*** Alternatively, other buffers (PBS, HBSS, culture medium, etc.) may also be used to store the harvested tissue.

***Note:*** If the harvested tissue will be processed with collagenase at Step 15, buffer without EDTA should be used because EDTA inhibits the enzymatic activity of collagenase.

### Part 4: Preparation of single-cell suspension

**Timing: 0.5–3 h**

**Figure 1:**
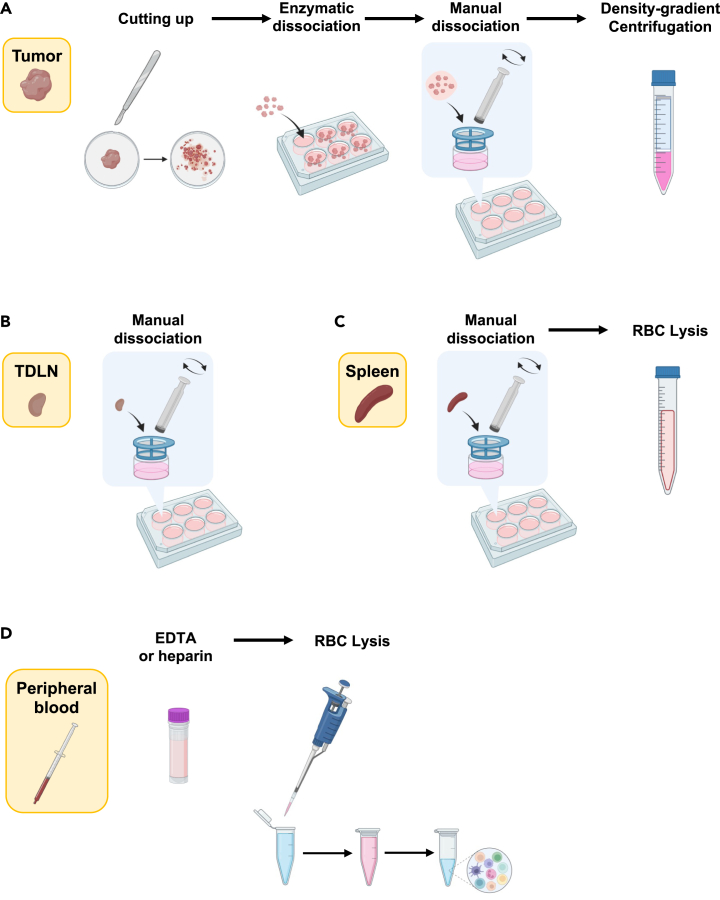
Workflow for preparation of single-cell suspensions Preparation of single-cell suspension for (A) tumor, (B) tumor-draining lymph node, (C) spleen, and (D) peripheral blood. Created with BioRender.com.

Single-cell suspensions are required for flow cytometry analysis and other omics assays. This section explains how to process different organs to obtain single-cell suspensions. (Figure 1).

### Tumor tissue

**Timing: 2–3 h**

***Note:*** Enzymatic tissue dissociation is required only for firm tumors (e.g. LLC lung carcinoma, MC38 colon carcinoma, 4T1 mammary cancer, etc.). Soft tumors (e.g. B16F10 melanoma) can skip this step and start from the manual tissue dissociation (Step 16).

***Note:*** Density-gradient centrifugation (Step 18) can be skipped when tumors do not contain much necrotic tissue.

***Note:*** In this protocol, we used collagenase D for enzymatic tissue dissociation followed by manual dissociation. Alternative options are available including gentleMACS Dissociator with Tumor dissociation kit (Miltenyi Biotec). However, some enzymes in the kit reagents alter the cell surface expression of certain proteins. Refer to the epitope preservation list before use. See also troubleshooting 1 and 2.

15. Enzymatic tissue dissociation (Figure 1A).
  a. In a petri dish, cut tumor tissue into small pieces (1–3 mm) with a scalpel or razor blade.
    ***Note:*** You may process half of the tumor for single-cell suspension and fix or snap-freeze the other half for histological analysis or any other desired assays.
  b. Transfer cut tissues into a 12-well plate. Add 2–3 mL of 1x collagenase D solution (1 mg/mL).
    ***Note:*** If tumor mass exceeds 500 mg, split tissue into 2 wells or use a 6-well plate.
  c. Incubate the tissue for 1 h at 37°C with gentle shaking.
    **CRITICAL:** You may need longer incubation times depending on the tumor type. However, over-incubation can damage and kill the cells.
  d. Transfer the digested tissue and solution to a 70 μm cell strainer placed in a new 6-well plate by using tip-cut transfer pipettes, serological pipettes, or wide-bore micropipette tips.
    ***Note:*** The cell strainers can be also placed over 50 mL centrifuge tubes instead of 6-well plates.
    ***Note:*** See also troubleshooting 1 and 2.
16. Manual tissue dissociation.
  a. Smash the tumor tissue against the 70 μm cell strainer using a syringe plunger to pass it through. Most of the tissue should go through the cell strainer.
  b. Transfer dissociated cell suspension into 15 mL centrifuge tubes.
  c. Centrifuge cells at 400–600 × g for 5 min at 20°C–25°C and discard the supernatant by aspiration or flicking of the tube.
17. Red blood cell (RBC) lysis.
  ***Note:*** The density-gradient centrifugation (Step 18) will also isolate RBCs. However, this step is recommended to clean up the single-cell suspension if the tumor tissues contain a lot of RBCs. Skip this step if the tumor tissues do not contain many RBCs.
  a. Resuspend cells in 3–5 mL of 1x ACK buffer.
  b. Incubate for 3–5 min at 20°C–25°C.
    ***Note:*** Incubation temperature can be either 20°C–25°C or on ice. Please follow the instructions from the manufacturer of the ACK buffer.
  c. Add PBS up to 15 mL to wash cells at 20°C–25°C.
  d. Centrifuge cells at 400–600 × g for 5 min at 20°C–25°C and discard the supernatant by aspiration or flicking of the tube.
18. Density-gradient centrifugation for debris removal.
  ***Note:*** See also troubleshooting 4.
  a. Resuspend cells in 8 mL of 40% Percoll solution.
  b. Insert a glass Pasteur pipet to the bottom of the 15 mL centrifuge tube containing the 40% Percoll cell suspension. Slowly layer 5 mL of 80% Percoll solution under 40% Percoll by slow addition through the glass Pasteur pipet. (Figure 2A).

**Figure 2:**
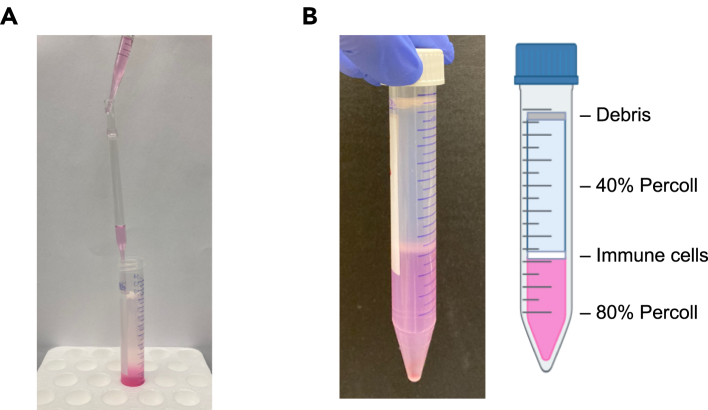
Percoll density-gradient centrifugation (A) Layering 80% Percoll solution under 40% Percoll solution using Pasteur pipet. (B) Density-gradient separation after centrifugation.
  c. Centrifuge at 600 × g for 20 min at 20°C–25°C with slow acceleration and no brake.
  d. Aspirate the layer of debris and 40% Percoll solution down to 1–2 cm above the interface.
    ***Note:*** The separation (interface) is visible after centrifugation as shown in Figure 2B.
  e. Transfer the immune cell-containing interface into a new 15 mL centrifuge tube with a transfer pipet.
  f. Add PBS up to 15 mL at 20°C–25°C.
  g. Centrifuge cells at 400–600 × g for 5 min at 20°C–25°C and discard the supernatant by aspiration or flicking of the tube.
19. Resuspend cells in flow cytometry buffer and keep cells on ice.
20. Determine cell number.

### Tumor-draining lymph node

**Timing: 30 min**

21. Manual tissue dissociation (Figure 1B).
  a. Smash the lymph nodes against the 70 μm cell strainer using a syringe plunger to pass it through into a 6-well plate containing 5–6 mL of flow cytometry buffer per well.
  b. Transfer cell suspension into 15 mL centrifuge tubes.
  c. Centrifuge cells at 400–600 × g for 5 min at 20°C–25°C and discard supernatant.
22. Resuspend cells in flow cytometry buffer and keep cells on ice.
23. Determine cell number.

### Spleen

**Timing: 30 min**

24. Manual tissue dissociation (Figure 1C).
  a. Smash the spleen against the 70 μm cell strainer using a syringe plunger to pass it through into a 6-well plate containing 5–6 mL of flow cytometry buffer per well.
  b. Transfer cell suspension into 15 mL centrifuge tubes.
  c. Centrifuge cells at 400–600 × g for 5 min at 20°C–25°C and discard supernatant.
25. RBC lysis.
  a. Resuspend cells in 3–5 mL of 1x ACK buffer.
  b. Incubate for 3–5 min at 20°C–25°C.
    ***Note:*** Incubation temperature can be either 20°C–25°C or on ice. Please follow the instructions from the manufacturer of the ACK buffer.
  c. Add PBS up to 15 mL to wash cells at 20°C–25°C.
  d. Centrifuge cells at 400–600 × g for 5 min at 20°C–25°C and discard supernatant.
26. Resuspend cells in flow cytometry buffer and keep cells on ice.
27. Determine cell number.

### Blood

**Timing: 30 min**

28. RBC lysis (Figure 1D).
  a. Add 30–50 μL of whole blood in 1.5 mL of 1x ACK buffer to a 1.5 mL microcentrifuge tube.
    ***Note:*** The volume of whole blood and 1x ACK buffer can be scaled up depending on the desired cell number.
  b. Incubate for 3–5 min at 20°C–25°C.
    ***Note:*** Incubation temperature can be either 20°C–25°C or on ice. Please follow the instructions from the manufacturer of the ACK buffer.
  c. Centrifuge cells at 400–600 × g for 5 min at 20°C–25°C and discard supernatant.
    ***Note:*** If you still see a red pellet at this step, it is acceptable to repeat Steps 28b and 28c. However, please keep in mind that over-incubation in ACK buffer can kill some cells. Even if a small amount of RBCs remains after a total of two rounds of Steps 28b and 28c, proceed to the next step to limit the loss of other cells.
  d. Resuspend cells in 1 mL of PBS for washing at 20°C–25°C.
  e. Centrifuge cells at 400–600 × g for 5 min at 20°C–25°C and discard supernatant.
29. Resuspend cells in flow cytometry buffer and keep cells on ice.
30. Determine cell number.

### Part 5: Staining of cells

**Timing: 3–4 h**

This section details how to stain cells with fluorescent-conjugated antibodies. Stained cells can be analyzed by flow cytometry. This protocol can also be used for cell sorting followed by cell population-specific single-cell RNA sequencing or other assays.

***Note:*** This protocol describes how to stain the cells in 96-well plate format. You can also stain cells in 5 mL flow cytometry tubes or 1.5 mL microcentrifuge tubes. When cells are stained in the tubes, a one-time wash with a larger volume of buffer should be enough to wash out reagents or excess antibodies. The wash steps throughout Part 5 can be modified as follows; add 1 mL of appropriate buffer (please follow the instructions at each step) to the tube, centrifuge, aspirate the supernatant, and move onward to the next step.

### Fixable live/dead staining

31. Plate 1 × 10^6^ cells per well in a v-bottom 96-well plate.
32. Wash cells with PBS.
  ***Note:*** Residual FBS or BSA will increase background signals.
  a. Add PBS up to 200 μL per well.
    ***Note:*** This protocol uses 96-well plates that can hold up to 350 μL/well. If 96-well plates with a lower maximum volume are being used, reduce the volume of PBS or other buffers used down to 2/3 of the maximum volume per well throughout the protocol.
  b. Centrifuge the plate at 400–600 × g for 3–5 min at 20°C–25°C.
  c. Discard the supernatant by flipping the plate.
33. Resuspend cells in 100 μL/well of Live/Dead staining solution. (See materials and equipment).
34. Incubate for 15 min at 20°C–25°C or 30 min on ice in the dark.
35. Wash the cells twice with PBS.
  a. Add 100 μL of PBS per well. (Total volume will be 100 μL per well).
  b. Centrifuge the plate at 400–600 × g for 3–5 min at 20°C–25°C.
  c. Discard the supernatant by flipping the plate.
  d. Resuspend the cells in 200 μL of PBS.
  e. Centrifuge the plate at 400–600 × g for 3–5 min at 20°C–25°C.
  f. Discard the supernatant by flipping the plate.

### Fc block

***Note:*** This step blocks non-specific antibody interaction mediated by Fc receptors mainly expressed on antigen-presenting cells (APCs) including macrophages, dendritic cells, and B cells.

36. Resuspend cells in 50 μL of Fc blocking solution. (See materials and equipment).
37. Incubate for 15 min on ice in the dark.

***Note:*** No washing steps are required after Fc blocking.

### Cell surface staining

38. Prepare an antibody master mix at 2x concentration in 50 μL per well in flow cytometry buffer during the Fc block incubation (Step 37). (See Tables 1 and 2 for example staining panels).

***Note:*** Brilliant Stain buffer is recommended when using Brilliant dyes to prevent interactions with Brilliant fluorescent polymer dyes.

***Note:*** See also troubleshooting 4.

39. Add 50 μL of 2x antibody master mix per well (Total volume will be 100 μL per well).
40. Incubate for 30 min on ice in the dark.
41. Wash the cells twice with flow cytometry buffer.
  a. Add 100 μL of flow cytometry buffer per well. (Total volume will be 100 μL per well).
  b. Centrifuge the plate at 400–600 × g for 3–5 min at 20°C–25°C.
  c. Discard the supernatant by flipping the plate.
  d. Resuspend the cells in 200 μL of flow cytometry buffer.
  e. Centrifuge the plate at 400–600 × g for 3–5 min at 20°C–25°C.
  f. Discard the supernatant by flipping the plate.
    ***Note:*** For single-cell sequencing analysis, specific populations (e.g. CD45^+^ immune cells or a specific population of immune cells) can be sorted after cell surface staining followed by library preparation (which is not described in this protocol). See also troubleshooting 5.

### Fixation

***Note:*** Skip this step if you do not need to stain intracellular proteins. Unfixed samples should be acquired on the day of the staining.

42. Prepare 1x Fixation buffer. (See materials and equipment).
43. Resuspend cells in 100 μL of Fixation buffer.
44. Incubate the cells for 30 min on ice in the dark.

**Pause point:** The samples can be in this fixation buffer for up to 16 hours at 4°C. Please follow the instructions from the manufacturer of the fixation buffer.

***Note:*** Alternatively, PFA can be used for fixation. Resuspend cells in 2% PFA/PBS and incubate the cells for 15 min on ice in the dark. The incubation time should not exceed 15 min because the PFA can damage the cells. PFA-fixation times can be variable depending on cell type and should be optimized.

### Permeabilization

***Note:*** If intracellular staining is not required, skip to Step 52 but wash with PBS instead of 1x permeabilization buffer.

45. Prepare 1x permeabilization buffer (See materials and equipment).
46. Centrifuge the plate at 800–1,000 × g for 3–5 min at 20°C–25°C.

***Note:*** Centrifuge at a higher speed after fixation because fixed cells become smaller.

47. Discard the supernatant by flipping the plate.
48. Wash the cells twice with 1x permeabilization buffer.
  a. Resuspend cells in 200 μL of 1x permeabilization buffer.
  b. Centrifuge the plate at 800–1,000 × g for 3–5 min at 20°C–25°C.
  c. Discard the supernatant by flipping the plate.
  d. Repeat 48 a–c, once.

### Intracellular staining

49. Prepare antibody master mix in 1x permeabilization buffer for intracellular staining. (See Tables 1 and 2 for example antibody panels).

***Note:*** See also troubleshooting 4.

50. Resuspend cells in 100 μL of intracellular staining antibody master mix.
51. Incubate for 30 min at 20°C–25°C in the dark.

***Note:*** See also troubleshooting 4.

52. Wash the cells twice with 1x permeabilization buffer.
  a. Add 100 μL of 1x permeabilization buffer per well. (Total volume will be 100 μL per well).
  b. Centrifuge the plate at 800–1,000 × g for 3–5 min at 20°C–25°C.
  c. Discard the supernatant by flipping the plate.
  d. Resuspend the cells in 200 μL of 1x permeabilization buffer.
  e. Centrifuge the plate at 800–1,000 × g for 3–5 min at 20°C–25°C.
  f. Discard the supernatant by flipping the plate.
53. Resuspend cells in an appropriate volume of flow cytometry buffer (150–200 μL).

### Preparation of fluorescence minus one (FMO) samples

***Note:*** FMO samples can be used to achieve proper gating, especially for the markers that do not show clear positive and negative peaks. Prepare multiple FMO samples for different fluorochromes as needed.

54. Prepare FMO samples for cell surface antigens by following the steps in Part 5 but exclude one antibody at Step 38.
55. Prepare FMO samples for intracellular antigens by following the steps in Part 5 but exclude one antibody at Step 49.

### Preparation of compensation beads

***Note:*** Single-color-stained samples are required for compensation optimization.

56. Prepare compensation beads (e.g., UltraComp eBeads Plus Compensation Beads) for fluorescent-conjugated antibodies according to the manufacturer’s protocol.
57. Prepare amine-reactive compensation beads (e.g., ArC Amine Reactive Compensation Bead Kit) for amine-reactive viability dyes according to the manufacturer’s protocol.

***Note:*** Cells may also be used for single-color-stained samples, but the use of compensation beads is preferred and recommended to obtain optimal compensation.

**Pause point:** The fixed samples can be stored at 4°C. It is recommended to acquire samples within 3 days of staining and fixation.

***Note:*** Some fluorochromes, especially tandem dyes, might degrade faster in storage. Signal stability needs to be tested before assuming that all signals are stable for 3 days.

### Part 6: Sample acquisition on a flow cytometer

**Timing: 1–4 h depending on the number of samples**

This section explains how to acquire stained samples and controls on a flow cytometer.

***Optional:*** Tumor samples can be passed through a 25–30 μm cell strainer immediately before sample acquisition. This will help to clean up the tumor samples and prevent clogging on the flow cytometer. (See also troubleshooting 3).

58. Adjust the voltage on each detector as needed.

***Note:*** This step is required for flow cytometers from BD or other companies. It is not usually required for a NovoCyte flow cytometer.

59. Perform automatic compensation.
  a. Acquire single-color stained compensation beads or samples for each fluorochrome.
    ***Note:*** For compensation beads, it is recommended to record a total of 10,000 events (5,000 events for both positive and negative populations). For cells, the number of events should be higher; at least 50,000 cells.
  b. Calculate a compensation matrix by using an automatic compensation function in the software.
60. Acquire FMO samples and a full-color-stained sample, then draw plots to identify different immune populations.

***Note:*** Use FMOs to set proper gating.

61. Once the flow cytometer has been properly configured, acquire all samples (wells).

***Note:*** Recording 100,000 events of the CD45^+^ population is recommended, however, this should be adjusted depending on your populations of interest. It is recommended to record at least 1,000 events of the population of interest. If the population is rare, you need to record 1 million or even more events of the CD45^+^ population accordingly.

62. Export your data in FCS file format.
63. Analyze the samples with FlowJo or a similar software. The gating strategies are shown in the Quantification and statistical analysis section.
64. Perform statistical analyses with GraphPad Prism software or similar.

## Expected outcomes

**Figure 3:**
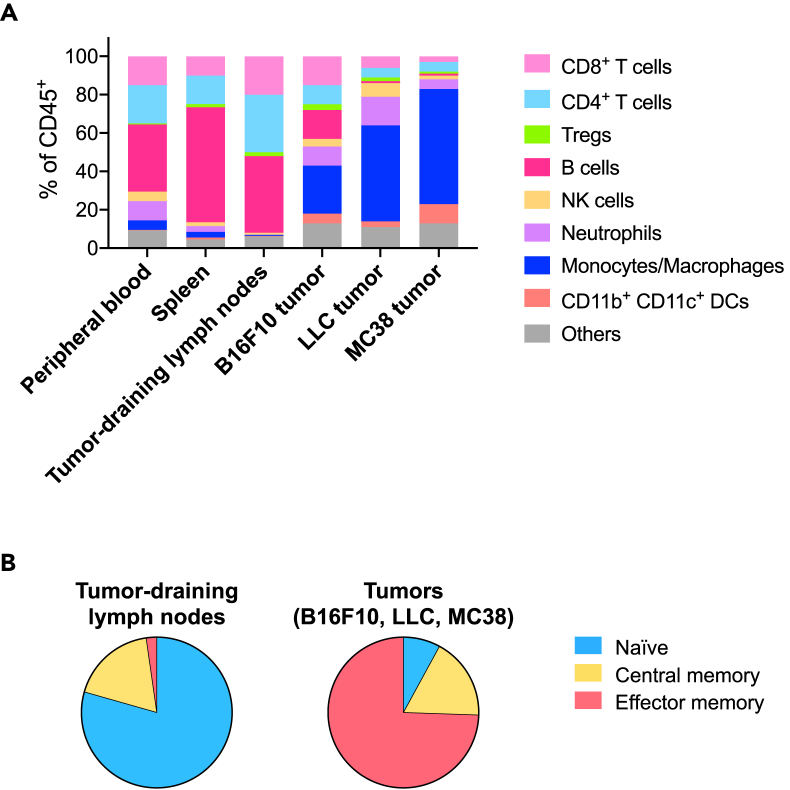
Immune profiling of different tissues (A) Immune profile of different tissues from tumor-bearing mice on Day 12. (B) Memory subsets of CD8^+^ T cells in tumor-draining lymph nodes and tumors.

The expected results of this protocol are shown in Figure 3.

Immune cell profiles are different depending on the organ or tissue (Figure 3A). Interestingly, different tumor types (cell lines) have distinct immune cell profiles. For example, LLC and MC38 tumors have myeloid cell-enriched profiles whereas B16F10 tumors have lymphoid cell-enriched profiles, indicating the uniqueness of the tumor microenvironment.

The development of memory T cell subsets in lymph nodes is a crucial step for the induction of antigen-specific anti-tumor immune response. The subsets of memory CD8^+^ T cells in tumor-draining lymph nodes and tumors are shown in Figure 3B. As expected, the majority of the CD8^+^ T cells in the tumor-draining lymph nodes are naïve and central memory subsets whereas most of the CD8^+^ T cells in the tumors are the effector memory subset, indicating the migration of the memory T cells developed in the tumor-draining lymph nodes into the tumor site.

Effects of treatments or your genes/proteins of interest on the tumor microenvironment can be investigated by analyzing the alterations in the immune cell profile induced by drug administration or gene knockout.

## Quantification and statistical analysis

The acquired events are first gated on FSC-A vs. SSC-A to remove debris and cell aggregations, then single cells are gated on FSC-A vs. FSC-H to remove doublets. Doublet discrimination can also be achieved by gating on FSC-H vs. FSC-W and then SSC-H vs. SSC-W. After excluding dead cells by gating viability dye-negative live cells, gate immune cells by CD45 positivity. The following strategies are used to identify different immune cell populations.

### Gating strategy for myeloid cells

**Figure 4:**
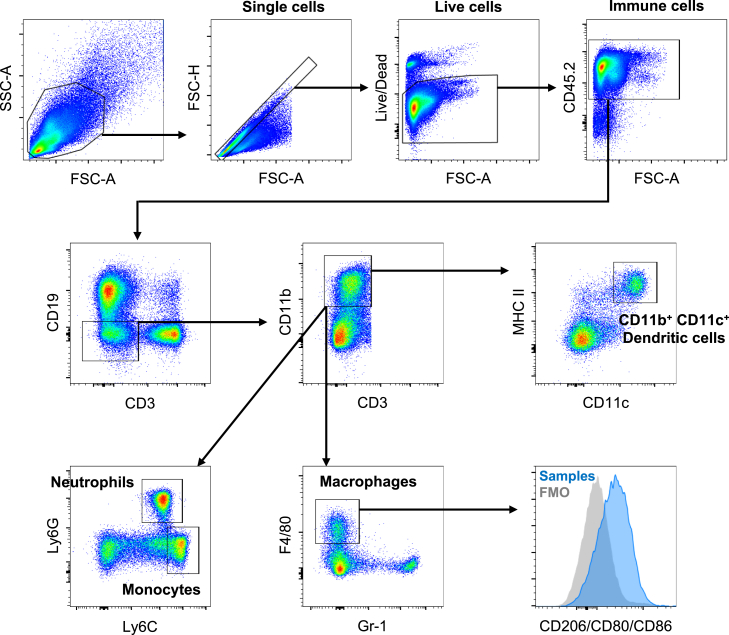
Gating strategy for myeloid cell populations Samples collected from the spleen are shown as an example.

After CD11b^+^ myeloid cell gating, neutrophils, monocytes, and macrophages can be defined with the following markers: neutrophils with Ly6G^+^/Ly6C^low^, monocytes with Ly6G^−^/Ly6C^high^, macrophages with F4/80^+^/Gr-1^−^ (Figure 4).^6^^,^^7^^,^^8^ The polarization and activation status of macrophages can be further analyzed with additional markers including CD206 for immunosuppressive macrophages and CD80/CD86 for proinflammatory macrophages.^9^^,^^10^

Some populations of dendritic cells can be identified with CD11b^+^/CD11c^+^/MHC II^+^, which includes monocyte-derived dendritic cells (mo-DCs) and conventional dendritic cells (cDC2).^11^^,^^12^ Additional markers are required to define cDC1, plasmacytoid DC (pDC), and migratory DC.

CD11b^+^/Ly6G^+^/Ly6C^low^ and CD11b^+^/Ly6G^−^/Ly6C^high^ include myeloid-derived suppressor cells (MDSC).^13^ However, functional markers need to be added to characterize the populations further.

### Gating strategy for lymphoid cells

**Figure 5:**
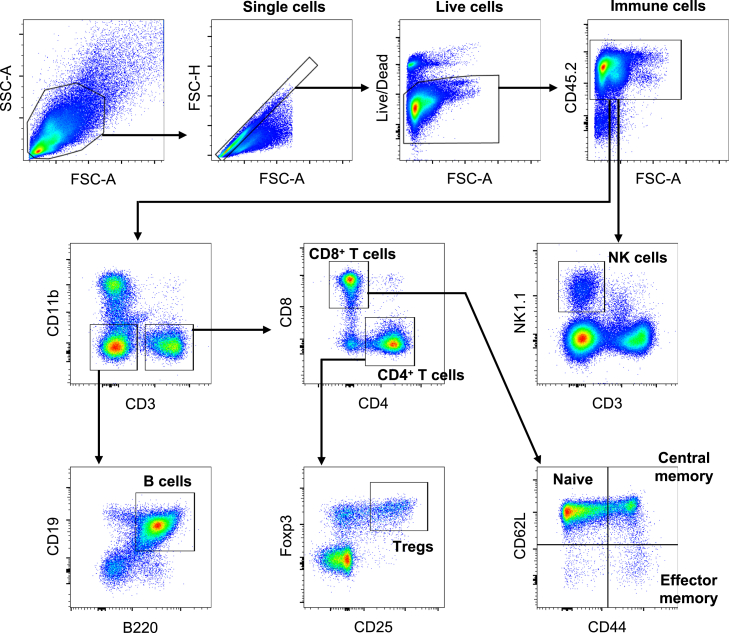
Gating strategy for lymphoid cell populations Samples collected from the spleen are shown as an example.

After CD3^+^ T cell gating, CD4^+^ T helper cells (CD4^+^/CD8^-^) and CD8^+^ cytotoxic T cells (CD8^+^/CD4^-^) can be identified.^7^ Regulatory T cells (Treg) can be identified as a CD25^+^/Foxp3^+^ subpopulation of CD4^+^ cells (Figure 5).^14^^,^^15^

Memory formation of T cells can be analyzed with a combination of CD62L and CD44; Naïve: CD62L^+^/CD44^-^, Central memory: CD62L^+^/CD44^+^, Effector memory: CD62L^-^/CD44^+^.^16^

B cells are a CD19^+^/B220^+^ population.^17^^,^^18^ To further classify the subpopulation of B cells including memory B cells, regulatory B cells, and plasma cells, additional markers must be used.

NK cells are an NK1.1^+^/CD3^-^ population whereas NK1.1^+^/CD3^+^ indicates NKT cells.^19^ NKG2D or other markers can be used to check activation status.

## Limitations

The staining panels in this study were designed to provide an immune profile overview, and the panels cover a majority of the main immune populations. Therefore, the information on their subpopulations is missing. For example, subsets of B cells (including memory B cells, regulatory B cells, and plasma cells) and subsets of dendritic cells (including plasmacytoid DCs and conventional DC1) cannot be distinguished by this staining panel. Also, functional markers need to be added to analyze the activation/exhaustion status or phenotype of some populations of immune cells including macrophages (anti-tumor or pro-tumor) and myeloid-derived suppressor cells (MDSCs) (monocytic-MDSC or polymorphonuclear-MDSC). The information can be obtained by adding/replacing markers specific to the cell types and cell states.

The configuration of the flow cytometer affects the results because each flow cytometer has a different spillover matrix. The designed staining panels need to be carefully tested.

## Troubleshooting

### Problem 1

After the enzymatic dissociation step, the tumor tissue does not pass through the cell strainer.

### Potential solution

Some tumor types require a longer incubation time for enzymatic reaction. Cutting tumors into smaller pieces to increase the surface area exposed to the enzyme solution could improve dissociation efficiency. Make sure to add enough enzymes. Adding more enzymes could help with efficient dissociation.

Also, using additional enzymes (e.g., hyaluronidase) could help the dissociation. However, some enzymes are known to affect the cell surface expression of proteins. Make sure the enzyme does not affect your proteins of interest.

If the cell suspension becomes viscous due to DNA release from digested cells, adding DNase I can alleviate this problem. Also, make sure the dissociation enzyme solution does not contain EDTA as it is a collagenase inhibitor. Using a bigger size of cell strainer (100 μm) before passing through smaller ones (40 or 70 μm) could also resolve the problem (Step 15).

### Problem 2

Some of the surface markers cannot be detected or lower frequencies than expected.

### Potential solution

Make sure to use appropriate dissociation enzymes. It has been reported that some dissociation enzymes decrease cell surface protein expression. For example, dispase decreases cell surface expression of CD8, which leads to a misreading of the immune profile^20^ (Step 15).

### Problem 3

The cells in single-cell suspension aggregate during the staining steps.

### Potential solution

Adding EDTA to buffers helps to prevent cell aggregation mediated by DNA released from dead cells. Passing suspension through a smaller size (25–30 μm) of cell strainer immediately before running samples on the flow cytometer could help remove cell aggregation and prevent clogging the lines in the flow cytometer (Step 58).

### Problem 4

The signal intensity of some markers is low.

### Potential solution

Make sure to add a sufficient concentration of antibodies. Adding more antibodies will help to get higher signals. It is recommended to titrate antibodies to find an optimal concentration. It can be different depending on the organs of interest (Steps 38 and 49).

If there are low intracellular marker signals, increasing the incubation time for permeabilization and intracellular staining steps could enhance the signals (Step 51).

If there are low tumor tissue signals, removing debris/necrotic tissue by Percoll gradient will help clean up your samples and improve the signal-to-noise ratio (Step 18).

### Problem 5

Infiltration of immune cells in the tumor is low and the frequency of immune cells in the single-cell suspension is too low to process for single-cell RNA sequence analysis or other assays.

### Potential solution

The frequency of immune cell infiltration depends on the immunogenicity of the tumor models. If the tumor type of interest is low immunogenicity and shows low immune cell infiltration, it might be better to sort CD45^+^ hematopoietic cell populations from tumor tissue. This will allow more sufficient numbers of immune cells to be analyzed and help you characterize the tumor microenvironment (Step 41).

## Resource availability

### Lead contact

Further information and requests for resources and reagents should be directed to and will be fulfilled by the lead contact, Dong-Er Zhang (d7zhang@health.ucsd.edu).

### Technical contact

Technical questions on executing this protocol should be directed to and will be answered by the technical contact, Sayuri Miyauchi (smiyauchi@health.ucsd.edu).

### Materials availability

This study did not generate new unique regents.

### Data and code availability

This study did not generate new datasets or report code.
